# Polygenic risk scores for atrial fibrillation and heart failure and the risk of stroke and dementia

**DOI:** 10.1093/braincomms/fcae477

**Published:** 2025-01-07

**Authors:** Lina Rydén, Nazib M Seidu, Hanna Wetterberg, Jenna Najar, Margda Waern, Silke Kern, Kaj Blennow, Henrik Zetterberg, Ingmar Skoog, Anna Zettergren

**Affiliations:** Department of Psychiatry and Neurochemistry, Institute of Neuroscience and Physiology, Sahlgrenska Academy, Centre for Ageing and Health (AgeCap) at the University of Gothenburg, Mölndal 43139, Sweden; Department of Psychiatry and Neurochemistry, Institute of Neuroscience and Physiology, Sahlgrenska Academy, Centre for Ageing and Health (AgeCap) at the University of Gothenburg, Mölndal 43139, Sweden; Department of Psychiatry and Neurochemistry, Institute of Neuroscience and Physiology, Sahlgrenska Academy, Centre for Ageing and Health (AgeCap) at the University of Gothenburg, Mölndal 43139, Sweden; Infection Medicine, Department of Clinical Sciences Lund, Lund University, Lund 22100, Sweden; Epidemiology, Population Studies and Infrastructures (EPI@LUND), Department of Laboratory Medicine, Lund University, Lund 22100, Sweden; Department of Psychiatry and Neurochemistry, Institute of Neuroscience and Physiology, Sahlgrenska Academy, Centre for Ageing and Health (AgeCap) at the University of Gothenburg, Mölndal 43139, Sweden; Region Västra Götaland, Sahlgrenska University Hospital, Psychiatry, Cognition and Old Age Psychiatry Clinic, Gothenburg 41346, Sweden; Section Genomics of Neurodegenerative Diseases and Aging, Department of Human Genetics Amsterdam UMC, Vrije Universiteit Amsterdam, Amsterdam UMC, Amsterdam 1081, The Netherlands; Department of Psychiatry and Neurochemistry, Institute of Neuroscience and Physiology, Sahlgrenska Academy, Centre for Ageing and Health (AgeCap) at the University of Gothenburg, Mölndal 43139, Sweden; Region Västra Götaland, Department of Psychotic Disorders, Sahlgrenska University Hospital, Gothenburg 41346, Sweden; Department of Psychiatry and Neurochemistry, Institute of Neuroscience and Physiology, Sahlgrenska Academy, Centre for Ageing and Health (AgeCap) at the University of Gothenburg, Mölndal 43139, Sweden; Region Västra Götaland, Sahlgrenska University Hospital, Psychiatry, Cognition and Old Age Psychiatry Clinic, Gothenburg 41346, Sweden; Paris Brain Institute, ICM, Pitié-Salpêtrière Hospital, Sorbonne University, Paris 75013, France; Neurodegenerative Disorder Research Center, Division of Life Sciences and Medicine, and Department of Neurology, Institute on Aging and Brain Disorders, University of Science and Technology of China and First Affiliated Hospital of USTC, Hefei 230026, China; Department of Psychiatry and Neurochemistry, Institute of Neuroscience and Physiology, The Sahlgrenska Academy at the University of Gothenburg, Mölndal 43180, Sweden; Clinical Neurochemistry Laboratory, Sahlgrenska University Hospital, Mölndal 43180, Sweden; Department of Psychiatry and Neurochemistry, Institute of Neuroscience and Physiology, The Sahlgrenska Academy at the University of Gothenburg, Mölndal 43180, Sweden; Clinical Neurochemistry Laboratory, Sahlgrenska University Hospital, Mölndal 43180, Sweden; Department of Neurodegenerative Disease, UCL Institute of Neurology, London WC1N 3BG, UK; UK Dementia Research Institute at UCL, London WC1E 6BT, UK; Hong Kong Center for Neurodegenerative Diseases, Clear Water Bay, Hong Kong SAR, China; Wisconsin Alzheimer’s Disease Research Center, University of Wisconsin School of Medicine and Public Health, University of Wisconsin-Madison, Madison, WI 53715, USA; Department of Psychiatry and Neurochemistry, Institute of Neuroscience and Physiology, Sahlgrenska Academy, Centre for Ageing and Health (AgeCap) at the University of Gothenburg, Mölndal 43139, Sweden; Region Västra Götaland, Sahlgrenska University Hospital, Psychiatry, Cognition and Old Age Psychiatry Clinic, Gothenburg 41346, Sweden; Department of Psychiatry and Neurochemistry, Institute of Neuroscience and Physiology, Sahlgrenska Academy, Centre for Ageing and Health (AgeCap) at the University of Gothenburg, Mölndal 43139, Sweden

**Keywords:** cardiovascular disease, neurologic disease, genetics, epidemiology

## Abstract

Atrial fibrillation and heart failure have both been suggested to increase stroke and dementia risk. However, in observational studies, reversed causation and unmeasured confounding may occur. To mitigate these issues, this study aims to investigate if higher genetic risk for atrial fibrillation and heart failure increases dementia and stroke risk. Data were obtained from the population-based Gothenburg H70 Birth Cohort Studies in Sweden. Participants (*N* = 984) were born in 1930 with baseline examinations at age 70, 75, 79 or 85 and follow-ups until age 88–89. Polygenic risk scores at the 5 × 10^−8^, 1 × 10^−5^, 1 × 10^−3^ and 1 × 10^−1^ thresholds were generated for atrial fibrillation and heart failure. Stroke was diagnosed based on self-reports, close-informant interviews, and the National Patient Register. Dementia was diagnosed based on neuropsychiatric examinations, close-informant interviews, and the National Patient Register. Cox regression analyses were performed, adjusted for sex, age at baseline and the first five principal components to correct for population stratification. Those within the highest atrial fibrillation-polygenic risk score tertile had a 1.5 (95% CI 1.09–2.03) increased risk of dementia (at the 1 × 10^−5^ threshold) and a 1.5 (95% CI 1.07–2.03) increased risk of stroke (at the 1 × 10^−3^ threshold) compared to the lowest tertile. Those within the highest heart failure-polygenic risk score tertile had a 1.6 (95% CI 1.19–2.27) increased risk of dementia (at the 5 × 10^−8^ threshold), but no increased risk of stroke (HR 1.2; 95% CI 0.83–1.60 at the 1 × 10^−5^ threshold), compared to the lowest tertile. When analysing the polygenic risk scores as a continuous variable, the associations were in the same direction, although weaker. This study, investigating genetic risk of atrial fibrillation and heart failure in relation to stroke and dementia, supports the increasing body of evidence suggesting that atrial fibrillation is associated with both stroke and dementia risk. Whether heart failure increases dementia risk is less established, but the present study found that genetic risk of heart failure increased dementia risk. The finding that genetic risk for heart failure did not increase stroke risk needs to be interpreted with caution, as it may be due to a lack of statistical power. There are guidelines on how to best treat atrial fibrillation to prevent stroke, but more knowledge is needed on how to treat atrial fibrillation and heart failure to prevent dementia.

## Teaser

Rydén *et al.* reported that higher genetic risk for AF (measured with PRSs) increases stroke and dementia risk and that higher genetic risk for HF increases dementia risk in the population-based Gothenburg H70 birth cohort study in Sweden.

## Graphical Abstract

**Graphical Abstract:**
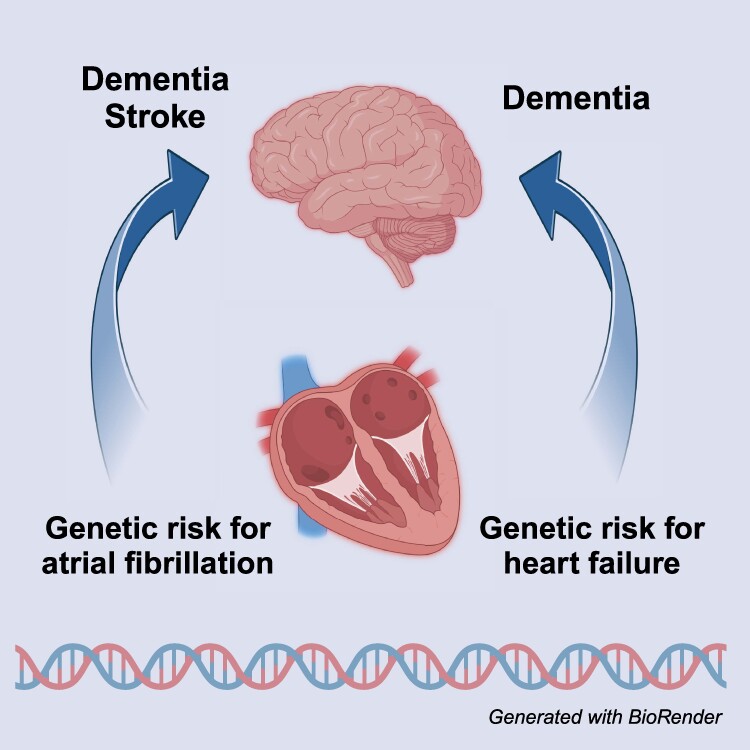

## Introduction

Stroke and dementia are major contributors to disability-adjusted life years (DALYs) and deaths worldwide.^1^ Even though the age-standardized rates of DALYs and deaths due to stroke and dementia have decreased during the last decade, the total numbers have increased due to an ageing and growing population, highlighting the need for further prevention of these two diseases.^1^ Atrial fibrillation (AF) is associated with a 5-fold increase in stroke risk.^2^ Stroke may, in turn, lead to dementia, but studies have shown that AF increases dementia risk also in individuals without stroke.^3,4^ Heart failure (HF) is a recognized risk factor for stroke in individuals with AF and is included in risk scores for predicting stroke risk in AF patients (e.g. the CHA_2_DS_2_-VASc score).^5^ Whether HF increases stroke risk independent of AF is less studied, although there are studies suggesting an association.^6-10^ Studies have also suggested an independent association between HF and dementia,^11-14^ with some exceptions.^15-17^

AF and HF have a bidirectional relationship and the co-existence of the two diseases increases the risk of adverse outcomes.^18^ Shared risk factors include hypertension, diabetes and obesity^19,20^ all of which are also associated with elevated risk of stroke^21^ and dementia.^22^ Even though common risk factors are included as potential covariates when studying the effect of AF and HF on stroke and dementia, there is a risk of unmeasured confounders. In addition, dementia has a long prodromal and preclinical phase, making it difficult to establish a causal relationship between a risk factor and dementia. One advantage of studying genetic risk of a predictor instead of the actual predictor (e.g. manifest diagnoses), is the possibility to overcome problems with unmeasured confounders and reverse causation.^23^

While AF can develop as a consequence of other diseases, such as HF or valvular disease, and environmental factors, a genetic component has also been reported.^24^ For example, the Framingham Heart Study found that individuals with a family history of AF had a 40% increased risk of developing the disease.^25^ HF has also been shown to have a genetic component, with a suggested heritability of around 30%.^26^ Since 2002, when the first genome-wide association study (GWAS) was published, linking a specific locus in the genome with myocardial infarction,^27,28^ GWASs have gained more attention. More recently, several two-sample Mendelian randomization (2SMR) studies, based on publicly available GWASs, have been conducted, investigating the association between genetic risk for AF or HF and genetic risk for stroke or dementia. 2SMR studies are cost-effective and convenient since they are based on publicly available GWAS data where the predictor and outcome do not have to be measured in the same sample. Regarding AF, 2SMR studies have shown a causal association with stroke,^29-35^ while other 2SMR studies focusing on the relationship between AF and dementia have yielded conflicting results.^36-38^ When it comes to HF, three 2SMR studies have shown an association between HF and any ischaemic stroke,^30,39,40^ but in two of these the relationship disappeared after adjusting for AF and coronary heart disease.^30,39^ In contrast, a 2SMR study on HF and dementia found a protective effect of HF on Alzheimer’s dementia.^41^

In addition to 2SMR studies, it has been shown that genetically predicted AF is associated with having a stroke diagnosis in case-control studies^42,43^ and incident stroke in longitudinal cohort studies.^44^ However, to the best of our knowledge, studies investigating if genetic risk for AF increases the risk of being diagnosed with dementia and if genetic risk for HF increase the risk of being diagnosed with stroke or dementia are lacking. Therefore, we aimed to investigate if higher polygenic risk scores (PRSs) for AF and HF increase the risk of stroke and dementia in a longitudinal population-based sample.

## Materials and methods

### Sample

Data were obtained from the population-based Gothenburg H70 Birth Cohort Studies (H70). The sample was systematically selected, based on birth dates from the Swedish population register, and includes individuals born in 1930 who lived in the municipally of Gothenburg at the time of the invitation. A detailed description of the cohort has been published elsewhere.^45,46^ Examinations were conducted at age 70–72 (year 2000–2002), 75–76 (year 2005–2007), 79–81 (year 2009–2011), 85–87 (year 2105–2017) and 88–89 (2018–2019). In total, 1094 individuals participated at least one time, of whom 984 have genetic data. Of those with genetic data, a total number of 564 had their baseline examination at age 70–72, 341 at age 75–76, 41 at age 79–81 and 38 at age 85–87.

### Data collection

The examinations included semi-structured interviews regarding somatic and psychiatric diseases and symptoms, medication use, and questions regarding social factors and lifestyle factors such as education. A physical examination was conducted, including a 12-lead ECG (coded according to the Minnesota Codes). A neuropsychiatric examination was performed including the mini-mental state examination^47^ and other tests of cognitive abilities (e.g. memory, proverbs, language, visuospatial and executive abilities, apraxia, construction and agnosia). Blood samples were drawn and analysed, e.g. glucose and cholesterol, and DNA was extracted. All participants were asked to provide contact information to a close informant and a close-informant interview was conducted including questions regarding somatic and psychiatric diseases and symptoms. Register data were obtained from the National Patient Register (NPR), including inpatient care and hospital-based outpatient care up to 2019. The inpatient part of the NPR started in 1964 and received full coverage of inpatient care in Gothenburg in 1973 (with the exception of year 1976).^48^ Since 1997, surgical day care was also included in the NPR and since 2001, all hospital-based in- and outpatient care was included.^49^ Death and emigration dates were obtained from the Swedish population register.

### Genotype data

Genotyping was performed with the NeuroChip (Illumina).^50^ During quality control (QC), participants were removed based on the following criteria: (i) Per-sample call rate <98%; (ii) Sex mismatch; (iii) High heterozygosity (defined as FHET (F coefficient estimate for assessing heterozygosity) outside ± 0.2); (iv) Non-European ancestral outliers (defined as the first two principal components (PCs) exceeding six standard deviations from the mean values of the European samples in the 1000 Genome global reference population); (v) Closely related samples based on pairwise PI_HAT (i.e. proportion of genome that are in identity-by-descent; calculated using –genome option in PLINK) ≥ 0.2; (vi) Per-single-nucleotide polymorphism (SNP) call rate < 98%, minor allele frequency < 0.01 and Hardy–Weinberg disequilibrium (*P* < 1 × 10^–6^). The Sanger imputation service was utilized to impute post-QC using the reference panel of Haplotype Reference Consortium data (HRC1.1).

### Polygenic risk scores

The AF-PRS was based on discovery data from a large and publicly available GWAS, including 60,620 cases and 970 216 controls of European ancestry.^51^ Five studies (The Nord-Trøndelag Health Study (HUNT), deCODE, the Michigan Genomics Initiative, DiscovEHR and the UK Biobank) and one consortium (the AFGen Consortium) including 31 cohorts,^52^ contributed to the GWAS.

The HF-PRS was also based on discovery data from a large and publicly available summarized GWAS, including 47 309 cases and 930 214 controls of European ancestry based on 26 studies from the Heart Failure Molecular Epidemiology for Therapeutic Targets Consortium.^53^

The SNPs for the PRSs were selected using four *P*-value thresholds, i.e. 5 × 10^−8^, 1 × 10^−5^, 1 × 10^−3^ and 1 × 10^−1^. In total, 138, 339, 2174 and 14830 SNPs were selected for the AF PRSs and 12, 78, 1545 and 13 023 SNPs were selected for the HF-PRSs, respectively. The PRSs were then calculated as the sum of the effect sizes (*β*-values) multiplied by the number of effect alleles of each SNP, per individual, in the target data.

### Diagnoses

*Dementia* was diagnosed according to the DSM-III-R criteria based on neuropsychiatric examinations, close-informant interviews and the NPR (ICD-8-SE codes: 290, 293.0 and 239.1; ICD-9-SE codes: 290, 294B, 331A, 331B, 331C and 331; ICD-10-SE codes: F00-02, F03.9, G30, G31.1 and G31.8A), as described in detail previously.^54^

*Stroke* was identified from self-reports and close-informant interviews, and the NPR (ICD8-SE codes 431, 433, 434; ICD9-SE codes 431, 432, 434, 438; ICD10-SE codes I61-I63, I69.1-I69.4). The criteria for a stroke diagnosis based on self- and close-informant interviews required a history of sudden onset of focal symptoms (including hemiparesis or aphasia) lasting for at least 24 h.

### Statistical analyses

Cox regression analyses were performed, adjusting for sex, age when first included in the study, and the first five PCs to correct for population stratification. Survival time was calculated from birth until an event occurred, the participant died, or the end of follow-up in 2019. The AF- and HF-PRSs were standardized and analysed as continuous variables and then divided into tertials, where those with the highest risk were compared to those with the lowest (referred to as ‘low’ and ‘high’ genetic risk) for further analyses. Individuals with missing data were omitted from the analyses. The proportional hazard assumption of the Cox regression models was tested with Schoenfeld’s residuals. Analyses were performed in R 4.3.2 using the survival and survminer packages. A two-tailed test with a *P*-value < 0.05 was considered significant.

## Ethical approval

The study was approved by the Regional Ethical Review Board in Gothenburg. Informed consent was obtained from all participants or their relatives if the participant was unable to provide informed consent.

## Results

In the total sample, including 984 participants (58.2% women), 250 (25.4%) were diagnosed with dementia (mean age at dementia onset: 80.3 years, SD 5.6) and the mean age at the end of follow-up was 84.4 years. In addition, 237 (24.2%) were diagnosed with stroke (mean age at stroke onset: 75.9 years, SD 9.6) and the mean age at the end of follow-up was 83.6 years. Sample characteristics stratified by dementia and stroke status are presented in Table 1.

**Table 1:** Sample characteristics

	Dementia	No dementia	Stroke	No stroke
	*n* = 250	*n* = 733	*n* = 238	*n* = 746
Females, *n* (%)	157 (62.8)	416 (56.8)	136 (57.1)	437 (58.6)
More than mandatory education, *n* (%)	113 (46.9)	343 (48.2)	99 (43.4)	357 (49.2)
AF, *n* (%)	110 (44.0)	271 (37.0)	119 (50.0)	236 (35.3)
Hospitalized for HF, *n* (%)	78 (31.2)	205 (28.0)	91 (38.2)	193 (25.9)
AF-PRS risk (5 × 10^−8^), *n* (%)				
High	91 (36.4)	237 (32.3)	81 (34.0)	247 (33.1)
Middle	82 (32.8)	245 (33.4)	87 (36.6)	241 (32.3)
Low	77 (30.8)	251 (34.2)	70 (29.4)	258 (34.6)
AF-PRS risk (1 × 10^−5^), *n* (%)				
High	97 (38.8)	230 (31.4)	93 (39.1)	235 (31.5)
Middle	78 (31.2)	250 (34.1)	72 (30.3)	256 (34.3)
Low	75 (30.0)	253 (34.5)	73 (30.7)	255 (34.2)
AF-PRS risk (1 × 10^−3^), *n* (%)				
High	92 (36.8)	236 (32.2)	91 (38.2)	237 (31.8)
Middle	78 (31.2)	249 (34.0)	77 (32.4)	251 (33.6)
Low	80 (32.0)	248 (33.8)	70 (29.4)	258 (34.6)
AF-PRS risk (1 × 10^−1^), *n* (%)				
High	80 (32.0)	247 (33.7)	80 (33.6)	248 (33.2)
Middle	86 (34.4)	242 (33.0)	87 (36.6)	241 (32.3)
Low	84 (33.6)	244 (33.3)	71 (29.8)	257 (34.5)
HF-PRS risk (5 × 10^−8^), *n* (%)				
High	96 (38.4)	231 (31.5)	80 (33.6)	248 (33.2)
Middle	89 (35.6)	239 (32.6)	75 (13.5)	253 (33.9)
Low	65 (26.0)	263 (35.9)	83 (34.9)	245 (32.8)
HF-PRS risk (1 × 10^−5^), *n* (%)				
High	90 (36.0)	238 (32.5)	81 (34.0)	247 (33.1)
Middle	92 (36.8)	236 (32.2)	88 (37.0)	240 (32.2)
Low	68 (27.2)	259 (35.3)	69 (29.0)	259 (34.7)
HF-PRS risk (1 × 10^−3^), *n* (%)				
High	84 (33.6)	243 (33.2)	76 (31.9)	252 (33.8)
Middle	84 (33.6)	244 (33.3)	80 (33.6)	248 (33.2)
Low	82 (32.8)	246 (33.6)	82 (34.5)	246 (33.0)
HF-PRS risk (1 × 10^−1^), *n* (%)				
High	92 (36.8)	235 (32.1)	86 (36.1)	242 (32.4)
Middle	83 (33.2)	245 (33.4)	70 (29.4)	258 (34.6)
Low	75 (30.0)	253 (34.5)	82 (34.5)	246 (33.0)

AF, atrial fibrillation; HF, heart failure; PRS, polygenic risk score.

Higher AF-PRS, as a continuous variable (Table 2), showed a trend toward an association with increased dementia risk at the 5 × 10^−8^ (hazard ratio (HR) 1.11, 95% CI 0.98–1.26, *P =* 0.093), 1 × 10^−5^ (HR 1.12, 95% CI 0.99–1.27, *P =* 0.071) and 1 × 10^−3^ (HR 1.12, 95% CI 0.99–1.28, *P =* 0.071) thresholds. In addition, higher AF-PRS increased the stroke risk at the 1 × 10^−3^ threshold (HR 1.14, 95% CI 1.01–1.31, *P =* 0.034) and the same trend was seen at the 1 × 10^−5^ threshold (HR 1.12, 95% CI 0.98–1.27, *P =* 0.086). Participants with higher HF-PRS (Table 2) had an increased dementia risk at the 5 × 10^−8^ threshold (HR 1.18, 95% CI 1.04–1.34, *P =* 0.010) and the same trend was seen at the 1 × 10^−5^ threshold (HR 1.13, 95% CI 0.99–1.28, *P =* 0.063). In contrast, participants with higher HF-PRS had no increased stroke risk.

**Table 2:** AF- and HF-PRSs in relation to dementia and stroke

	Dementia		Stroke	
	HR (95% CI)	*P*	HR (95% CI)	*P*
AF-PRS 5 × 10^−8^	1.11 (0.98–1.26)	0.093	1.10 (0.97–1.25)	0.136
AF-PRS 1 × 10^−5^	1.12 (0.99–1.27)	0.071	1.12 (0.98–1.27)	0.086
AF-PRS 1 × 10^−3^	1.12 (0.99–1.28)	0.071	1.14 (1.01–1.31)	0.034
AF-PRS 1 × 10^−1^	1.01 (0.89–1.15)	0.866	1.08 (0.95–1.23)	0.233
HF-PRS 5 × 10^−8^	1.18 (1.04–1.34)	0.010	1.01 (0.88–1.15)	0.903
HF-PRS 1 × 10^−5^	1.13 (0.99–1.28)	0.063	1.08 (0.95–1.24)	0.221
HF-PRS 1 × 10^−3^	1.01 (0.88–1.16)	0.835	0.97 (0.85–1.12)	0.686
HF-PRS 1 × 10^−1^	1.11 (0.97–1.28)	0.116	1.01 (0.88–1.17)	0.860

Analyses are adjusted for sex, age when first included in the study and the first five PCs.

AF, atrial fibrillation; HF, heart failure; HR, hazard ratio; PRS, polygenic risk score.

**Figure 1:**
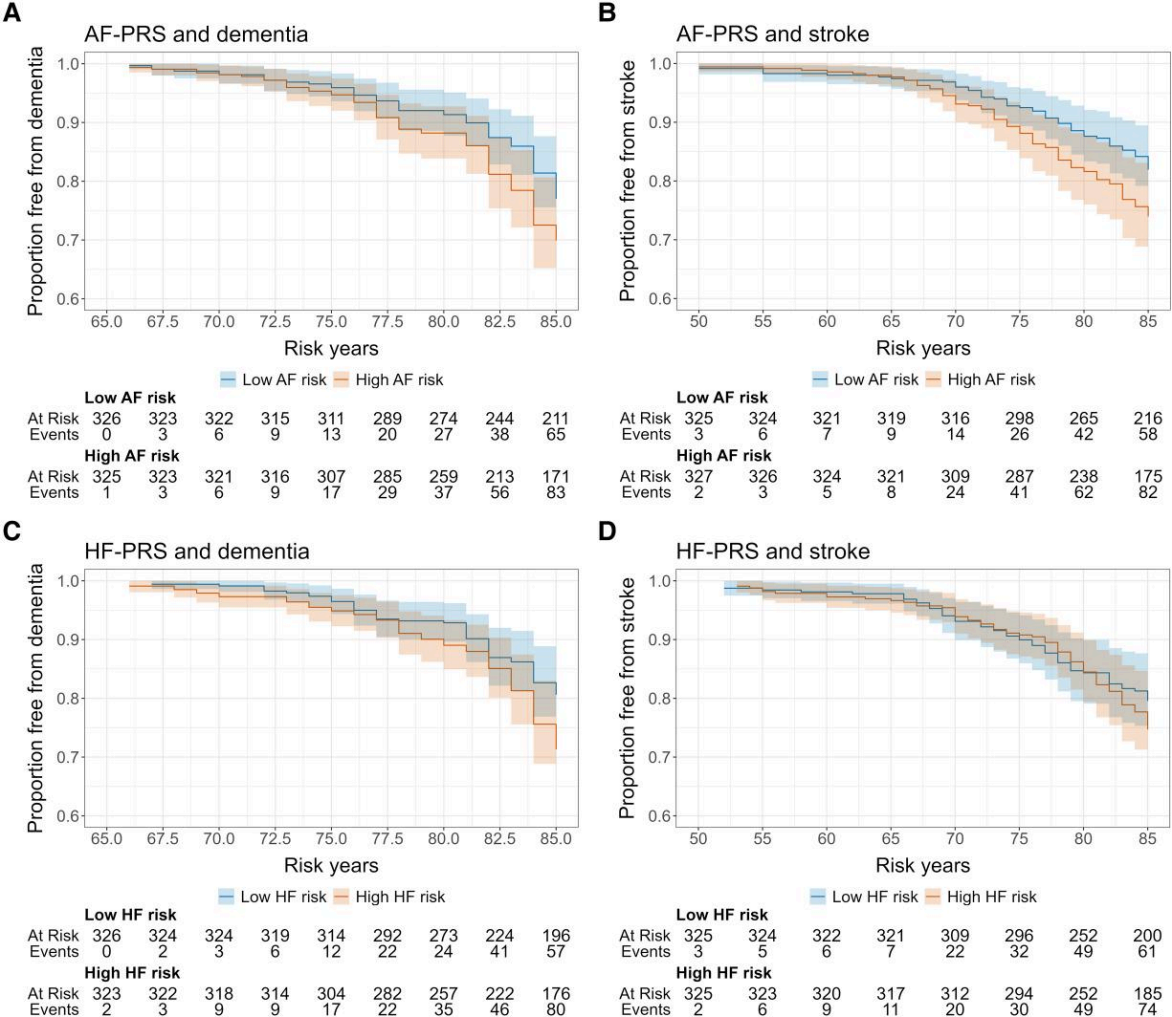
**Genetic risk for AF and HF in relation to dementia and stroke.** Participants with ‘high’ compared with ‘low’ genetic risk for (**A**) AF and the risk of dementia (Cox regression, HR *=* 1.49, 95% CI 1.09–2.03, *Z* = 2.52*, P =* 0.012), (**B**) AF and the risk of stroke (Cox regression, HR = 1.48, 95% CI 1.07*–*2.03, Z = 2.41, *P* = 0.016), (**C**) HF and the risk of dementia (Cox regression, HR = 1.64, 95% CI 1.19–2.27, *Z* = 3.02, *P* = 0.003) and (**D**) HF and the risk of stroke (Cox regression, HR = 1.15, 95% CI 0.83–1.60, *Z =* 0.85, *P =* 0.396). The PRSs with the strongest association to the outcome are shown (i.e. (**A**) 1 × 10^−5^, (**B**) 1 × 10^−3^, (**C**) 5 × 10^−8^ and (**D**) 1 × 10^−5^).

When dividing the PRSs into tertiles and comparing those in the highest tertile with those in the lowest, the same pattern occurred, although the associations were generally stronger. Participants with the highest AF-PRS at the 1 × 10^−5^ threshold had a 1.49 (95% CI 1.09–2.03, *P =* 0.012) increased risk for dementia (Table 3 and Fig. 1A) and the same trend was seen at the 1 × 10^−3^ threshold (HR 1.35, 95% CI 0.99–1.84, *P =* 0.056). Those with the highest AF-PRS at the 1 × 10^−3^ and 1 × 10^−5^ thresholds had a 1.48 (95% CI 1.07–2.03, *P =* 0.016) and 1.43 (95% CI 1.04–1.96, *P =* 0.024) increased risk for stroke, respectively (Table 3 and Fig. 1B).

**Table 3:** Highest AF- and HF-PRS tertials compared to the lowest in relation to dementia and stroke

	Dementia		Stroke	
	HR (95% CI)	*P*	HR (95% CI)	*P*
AF-PRS 5 × 10^−8^	1.26 (0.92–1.72)	0.143	1.20 (0.86–1.66)	0.276
AF-PRS 1 × 10^−5^	1.49 (1.09–2.03)	0.012	1.43 (1.04–1.96)	0.024
AF-PRS 1 × 10^−3^	1.35 (0.99–1.84)	0.056	1.48 (1.07–2.03)	0.016
AF-PRS 1 × 10^−1^	0.96 (0.70–1.31)	0.787	1.21 (0.87–1.67)	0.258
HF-PRS 5 × 10^−8^	1.64 (1.19–2.27)	0.003	0.98 (0.71–1.34)	0.886
HF-PRS 1 × 10^−5^	1.45 (1.05–2.01)	0.024	1.15 (0.83–1.60)	0.396
HF-PRS 1 × 10^−3^	1.08 (0.78–1.49)	0.629	0.93 (0.67–1.29)	0.674
HF-PRS 1 × 10^−1^	1.31 (0.95–1.79)	0.096	1.05 (0.77–1.44)	0.747

Analyses are adjusted for sex, age when first included in the study, and the first five PCs.

AF, atrial fibrillation; HF, heart failure; HR, hazard ratio; PRS, polygenic risk score.

Participants with the highest HF-PRS at the 1 × 10^−5^ and 5 × 10^−8^ thresholds had a 1.45 (95% CI 1.05–2.01, *P =* 0.024) and 1.64 (95% CI 1.19–2.27, *P =* 0.003) increased risk for dementia, respectively, and the same trend was seen at the 1 × 10^−1^ threshold (HR 1.31, 95% CI 0.95–1.79, *P =* 0.096) (Table 3 and Fig. 1C). Participants with the highest HF-PRS had no increased stroke risk, compared with those with the lowest risk (Table 3 and Fig. 1D).

Due to problems with influential observations among participants included at ages 79 and 85, the analyses were repeated after exclusion of all individuals entering the study at ages 79 and 85. This did not change the results. In the analyses including stroke, sex had non-proportional hazards, and analyses were thus repeated after stratifying for sex. For men, the results were in the same direction as for the total group, even though the association between higher AF-PRS as a continuous variable and stroke was no longer significant for the AF-PRS at the 1 × 10^−3^ threshold (men: HR 1.2, *P =* 0.083; total group: HR 1.1, *P =* 0.034). In contrast, when dividing the PRSs into tertiles and comparing those with the highest AF risk with those with the lowest risk, the stroke risk was still increased for the AF-PRS at the 1 × 10^−5^ threshold (men: HR 2.2, *P =* 0.003, total group: HR 1.4 *P =* 0.024) and the 1 × 10^−3^ threshold (men: HR 1.7, *P =* 0.029; total group: HR 1.5, *P =* 0.016). For women, no increased risk of stroke was seen.

## Discussion

Those within the highest AF-PRS tertile (at the 1 × 10^−3^ and 1 × 10^−5^ thresholds) had a 1.4–1.5 increased risk of dementia and stroke compared with those within the lowest. Those within the highest HF-PRS tertile (at the 1 × 10^−5^ and 5 × 10^−8^ thresholds) had a 1.5–1.6 increased risk of dementia, but no increased risk of stroke, compared to those within the lowest.

### AF and dementia

The finding that those with the highest AF-PRS had an increased dementia risk compared to those with the lowest is in line with a large body of longitudinal observation studies, suggesting an increased dementia risk in individuals with an AF diagnosis.^4,55-60^ Our results contrast with those of three previous 2SMR studies showing that genetic risk for AF did not increase the risk of vascular dementia^36^ or Alzheimer’s dementia.^33,38^ However, in the present study, we did not study specific dementia types and a recently published 2SMR study found that genetically predicted AF was associated with all-cause dementia.^37^ The association in this 2SMR study disappeared after adjusting for genetically predicted stroke or low cardiac output, suggesting that these factors might be mediators in the possible association between AF and dementia. Another possible mechanism behind an association between AF and dementia involves inflammation. It has, for example, been shown that C-reactive protein levels decrease in patients who remain in sinus rhythm after ablation or cardioversion, but not in patients with recurrent AF.^61^ It has also been discussed whether brain abnormalities might trigger AF (i.e. a reversed causation). One example of a possible mechanism is that higher Abeta levels have been found in the myocardium of patients with Alzheimer’s dementia compared to controls,^62^ which may affect cardiac cell function.

### AF and stroke

That genetically predicted AF is associated with stroke is in line with a community-based study from the Malmö Diet and Cancer Study in Sweden, which used a 12 SNP AF-PRS and found a 23% increase in risk of ischaemic stroke in individuals with the highest genetic risk for AF compared with those with the lowest risk.^44^ Furthermore, a case-control study found an association between AF PRSs based on 172 to 162 456 SNPs and cardioembolic stroke, but not between AF and other ischaemic stroke subtypes (i.e. large vessel, small vessel, or unknown subtypes).^42^ A third genetic study, using an AF-PRS constructed of 934 SNPs, found a strong association with cardioembolic stroke, also after adjustment for clinical risk factors.^63^ That AF increases the risk of any stroke,^29,30,33,35^ ischaemic stroke^29-35^ and cardioembolic stroke^29,30,33-35^ is also supported by a number of 2SMR studies. AF may increase the risk for cardiac embolism to the brain by causing abnormal blood flow, abnormalities of the vessel walls and hypercoagulability (i.e. the Virchow’s triad), rendering a causal association between AF and ischaemic stroke likely. However, two of the 2SMR studies found a bidirectional relationship between AF and ischaemic stroke^31,33^ and stroke might increase AF risk due to an impact on the autonomic nervous system and an inflammatory response from the brain injury.^64^

### HF and dementia

Our finding that higher HF-PRS increases dementia risk is in line with several longitudinal observation studies showing a higher risk to develop all-cause dementia for individuals with an HF diagnosis.^11-14^ In contrast, a recent meta-analysis did not find an association between HF diagnoses and dementia.^65^ However, this meta-analysis included one large register-based study from Sweden studying the relationship between HF and dementia in AF patients. When leaving this study out, the meta-analysed studies showed an almost 1.3 times increased risk of dementia in participants with HF compared to those without HF.^65^ One plausible mechanism behind a possible association between HF and dementia is reduced cardiac output.^66,67^ In addition, HF has been associated with inflammation and oxidative stress.^68^ Regarding evidence from studies on genetically predicted HF, two 2SMR studies found that genetically predicted HF did not increase Alzheimer’s dementia risk,^41,69^ in fact, they supported a reversed association, i.e. that HF is protective of Alzheimer’s dementia. In the present study, we have only investigated all-cause dementia and it is possible that the association between HF-PRS and all-cause dementia is driven by other dementia types than Alzheimer’s dementia. This is supported by one large register-based study from Denmark, which found no effect of HF on Alzheimer’s dementia while there was a 1.5 increased risk of vascular dementia in individuals with HF.^11^ The protective effect of genetically predicted HF seen in the 2SMR studies has been suggested to be related to the protective effects of HF drugs on Alzheimer’s dementia or the competing risk of death in patients with HF.^41^

### HF and stroke

Our negative finding regarding HF and stroke was somewhat unexpected as register-based studies have found an increased risk for ischaemic stroke,^6,7,9,10^ intracranial haemorrhage^6,7,10^ and subarachnoid haemorrhage.^7,10^ Also the population-based Rotterdam study has found an increased stroke risk in HF patients, especially during the first month after an HF diagnosis.^8^ But the increased risk disappeared over time with no increased risk 0.5–6 years after the diagnosis. 2SMR studies have also found an association between HF and any stroke^30^ and ischaemic stroke.^30,39,40^ However, in two of the studies,^30,39^ the associations disappeared after adjustment for AF and coronary heart disease. In the third study, the association remained after adjustments.^40^ This study also investigated if there was a bidirectional relation and found that stroke also increased HF risk. However, this relation disappeared after adjustment for coronary heart disease.^40^ That higher genetic risk for HF did not increase stroke risk in the present study needs to be interpreted with caution, as it may be due to a lack of statistical power. Similar to AF, HF has been suggested to cause thromboembolism in the context of Virchow’s triad, where reduced cardiac output, dilated cardiac chambers and poor contractility of the chambers result in abnormal blood flow.^70^ Since AF often goes unrecognized in individuals due to its paroxysmal and sometimes asymptomatic nature, the association seen between HF diagnoses and stroke in register and population-based studies might be confounded by ‘silent’ AF, which is known to increase the risk for stroke. The third 2SMR study^40^ did, however, find an association even after adjusting for genetically predicted AF.

### Strengths and limitations

Strengths of the study include the possibility to test associations between genetic markers and disease outcomes in a population-based sample, which increases the possibility to generalize the results to the general population, at least to genetically similar populations. Genetic studies often require large sample sizes, but by calculating PRSs based on results from GWASs, effects may be shown also in smaller cohorts. In addition, both stroke and dementia diagnoses are based on extensive clinical examinations and register data. Some limitations also need to be addressed. First, due to the relatively small sample size, negative associations need to be interpreted with caution, and we could not investigate associations with dementia subtypes. Furthermore, we could not distinguish between haemorrhagic and ischaemic stroke, and it is possible that AF and HF are associated with specific stroke subtypes. Second, the first examination point was at age 70, and therefore diagnoses identified before this age rely on retrospective reports and hospital inpatient data. The hospital register has low coverage when it comes to dementia,^71^ which explains why diagnoses can be missed. However, since dementia is uncommon in younger ages, this is likely not a big concern. The fact that the first examination was at age 70 also means that participants in the study must survive to age 70 to be included.

### Conclusion

Our finding that AF and HF increase dementia risk expands on the knowledge from previous non-genetic observational studies. The present study suggests a 50% increased risk of dementia for those with the highest genetic risk for AF and a 60% increased risk for those with the highest genetic risk for HF compared to those with the lowest risk. We also found a 50% increased risk of stroke for those with the highest genetic risk for AF, but no increased stroke risk in those with the highest genetic HF risk compared to those with the lowest risk. Today, stroke risk is assessed in patients with AF, which guides treatment choices. However, more knowledge is needed on how to best assess dementia risk in both AF and HF patients as well as the best treatment regimens in these patient groups to prevent dementia.

## Data Availability

The data that support the findings of this study are available from the corresponding author, upon reasonable request. The codes used for statistical analyses are openly available in zenodo at http://doi.org/10.5281/zenodo.14194472.

## References

[1] Avan A , Hachinski V. Stroke and dementia, leading causes of neurological disability and death, potential for prevention. Alzheimers Dement. 2021;17(6):1072–1076. 34057294 10.1002/alz.12340

[2] Virani SS , Alonso A, Benjamin EJ, et al Heart disease and stroke statistics-2020 update: A report from the American Heart Association. Circulation. 2020;141(9):e139–e596. 31992061 10.1161/CIR.0000000000000757

[3] Kim D , Yang PS, Yu HT, et al Risk of dementia in stroke-free patients diagnosed with atrial fibrillation: Data from a population-based cohort. Eur Heart J. 2019;40(28):2313–2323. 31212315 10.1093/eurheartj/ehz386

[4] Rydén L , Zettergren A, Seidu NM, et al Atrial fibrillation increases the risk of dementia amongst older adults even in the absence of stroke. J Intern Med. 2019;286(1):101–110. 30895641 10.1111/joim.12902

[5] Hindricks G , Potpara T, Dagres N, et al 2020 ESC guidelines for the diagnosis and management of atrial fibrillation developed in collaboration with the European association for cardio-thoracic surgery (EACTS): The task force for the diagnosis and management of atrial fibrillation of the European Society of Cardiology (ESC) developed with the special contribution of the European heart rhythm association (EHRA) of the ESC. Eur Heart J. 2021;42(5):373–498. 32860505 10.1093/eurheartj/ehaa612

[6] Chou Y-L , Liou J-T, Cheng C-C, et al The association of ischaemic stroke in patients with heart failure without atrial flutter/fibrillation. Heart. 2020;106(8):616–623. 31582568 10.1136/heartjnl-2019-315646

[7] Adelborg K , Szépligeti S, Sundbøll J, et al Risk of stroke in patients with heart failure: A population-based 30-year cohort study. Stroke. 2017;48(5):1161–1168. 28377383 10.1161/STROKEAHA.116.016022

[8] Alberts VP , Bos MJ, Koudstaal P, et al Heart failure and the risk of stroke: The Rotterdam study. Eur J Epidemiol. 2010;25(11):807–812. 21061046 10.1007/s10654-010-9520-y PMC2991556

[9] Berger JS , Peterson E, LalibertÉ F, et al Risk of ischemic stroke in patients newly diagnosed with heart failure: Focus on patients without atrial fibrillation. J Card Fail. 2019;25(6):436–447. 29597052 10.1016/j.cardfail.2018.03.012

[10] Tai YH , Chang CC, Yeh CC, et al Long-term risk of stroke and poststroke outcomes in patients with heart failure: Two nationwide studies. Clin Epidemiol. 2020;12:1235–1244. 33177880 10.2147/CLEP.S261179 PMC7652062

[11] Adelborg K , Horváth-Puhó E, Ording A, Pedersen L, Sørensen HT, Henderson VW. Heart failure and risk of dementia: A Danish nationwide population-based cohort study. Eur J Heart Fail. 2017;19(2):253–260. 27612177 10.1002/ejhf.631 PMC5522185

[12] Legdeur N , van der Lee SJ, de Wilde M, et al The association of vascular disorders with incident dementia in different age groups. Alzheimers Res Ther. 2019;11(1):47. 31097030 10.1186/s13195-019-0496-x PMC6524321

[13] Qiu C , Winblad B, Marengoni A, Klarin I, Fastbom J, Fratiglioni L. Heart failure and risk of dementia and Alzheimer disease: A population-based cohort study. Arch Intern Med. 2006;166(9):1003–1008. 16682574 10.1001/archinte.166.9.1003

[14] Rusanen M , Kivipelto M, Levälahti E, et al Heart diseases and long-term risk of dementia and Alzheimer’s disease: A population-based CAIDE study. J Alzheimers Dis. 2014;42(1):183–191. 24825565 10.3233/JAD-132363

[15] Haring B , Leng X, Robinson J, et al Cardiovascular disease and cognitive decline in postmenopausal women: Results from the women’s health initiative memory study. J Am Heart Assoc. 2013;2(6):e000369. 24351701 10.1161/JAHA.113.000369 PMC3886762

[16] de Bruijn RF , Bos MJ, Portegies ML, et al The potential for prevention of dementia across two decades: The prospective, population-based Rotterdam study. BMC Med. 2015;13:132. 26195085 10.1186/s12916-015-0377-5 PMC4509699

[17] Peters R , Poulter R, Beckett N, et al Cardiovascular and biochemical risk factors for incident dementia in the hypertension in the very elderly trial. J Hypertens. 2009;27(10):2055–2062. 19696686 10.1097/HJH.0b013e32832f4f02

[18] Kornej J , Börschel CS, Benjamin EJ, Schnabel RB. Epidemiology of atrial fibrillation in the 21st century: Novel methods and new insights. Circ Res. 2020;127(1):4–20. 32716709 10.1161/CIRCRESAHA.120.316340 PMC7577553

[19] Allan V , Honarbakhsh S, Casas JP, et al Are cardiovascular risk factors also associated with the incidence of atrial fibrillation? A systematic review and field synopsis of 23 factors in 32 population-based cohorts of 20 million participants. Thromb Haemost. 2017;117(5):837–850. 28229164 10.1160/TH16-11-0825 PMC5442605

[20] Triposkiadis F , Xanthopoulos A, Parissis J, Butler J, Farmakis D. Pathogenesis of chronic heart failure: Cardiovascular aging, risk factors, comorbidities, and disease modifiers. Heart Fail Rev. 2022;27(1):337–344. 32524327 10.1007/s10741-020-09987-z

[21] Hankey GJ . Stroke. Lancet. 2017;389(10069):641–654. 27637676 10.1016/S0140-6736(16)30962-X

[22] Livingston G , Huntley J, Sommerlad A, et al Dementia prevention, intervention, and care: 2020 report of the lancet commission. Lancet. 2020;396(10248):413–446. 32738937 10.1016/S0140-6736(20)30367-6 PMC7392084

[23] Lawlor DA , Harbord RM, Sterne JA, Timpson N, Davey Smith G. Mendelian randomization: Using genes as instruments for making causal inferences in epidemiology. Stat Med. 2008;27(8):1133–1163. 17886233 10.1002/sim.3034

[24] Christophersen IE , Ellinor PT. Genetics of atrial fibrillation: From families to genomes. J Hum Genet. 2016;61(1):61–70. 25994868 10.1038/jhg.2015.44

[25] Lubitz SA , Yin X, Fontes JD, et al Association between familial atrial fibrillation and risk of new-onset atrial fibrillation. JAMA. 2010;304(20):2263–2269. 21076174 10.1001/jama.2010.1690 PMC3073054

[26] Lindgren MP , PirouziFard M, Smith JG, Sundquist J, Sundquist K, Zöller B. A Swedish nationwide adoption study of the heritability of heart failure. JAMA Cardiol. 2018;3(8):703–710. 29998296 10.1001/jamacardio.2018.1919 PMC6583873

[27] Ozaki K , Ohnishi Y, Iida A, et al Functional SNPs in the lymphotoxin-alpha gene that are associated with susceptibility to myocardial infarction. Nat Genet. 2002;32(4):650–654. 12426569 10.1038/ng1047

[28] Ikegawa S . A short history of the genome-wide association study: Where we were and where we are going. Genomics Inform. 2012;10(4):220–225. 23346033 10.5808/GI.2012.10.4.220 PMC3543921

[29] Chen L , Peters JE, Prins B, et al Systematic Mendelian randomization using the human plasma proteome to discover potential therapeutic targets for stroke. Nat Commun. 2022;13(1):6143. 36253349 10.1038/s41467-022-33675-1 PMC9576777

[30] Frerich S , Malik R, Georgakis MK, et al Cardiac risk factors for stroke: A comprehensive Mendelian randomization study. Stroke. 2022;53(4):e130–e135. 34911345 10.1161/STROKEAHA.121.036306 PMC10510836

[31] Hou L , Xu M, Yu Y, et al Exploring the causal pathway from ischemic stroke to atrial fibrillation: A network Mendelian randomization study. Mol Med. 2020;26(1):7. 31941463 10.1186/s10020-019-0133-y PMC6964084

[32] Hu M , Tan J, Yang J, Gao X, Yang Y. Use of Mendelian randomization to evaluate the effect of atrial fibrillation on cardiovascular diseases and cardiac death. ESC Heart Fail. 2023;10(1):628–636. 36404673 10.1002/ehf2.14237 PMC9871698

[33] Kwok MK , Schooling CM. Mendelian randomization study on atrial fibrillation and cardiovascular disease subtypes. Sci Rep. 2021;11(1):18682. 34548541 10.1038/s41598-021-98058-w PMC8455674

[34] Li HQ , Feng YW, Yang YX, et al Causal relations between exposome and stroke: A Mendelian randomization study. J Stroke. 2022;24(2):236–244. 35677978 10.5853/jos.2021.01340 PMC9194538

[35] Tian D , Zhang L, Zhuang Z, Huang T, Fan D. A Mendelian randomization analysis of the relationship between cardioembolic risk factors and ischemic stroke. Sci Rep. 2021;11(1):14583. 34272412 10.1038/s41598-021-93979-y PMC8285403

[36] Gao YF , Jin TY, Chen Y, Ding YH. No causal genetic relationships between atrial fibrillation and vascular dementia: A bidirectional Mendelian randomization study. Front Cardiovasc Med. 2023;10:1071574. 37456823 10.3389/fcvm.2023.1071574 PMC10347408

[37] Li M , Jiang C, Lai Y, et al Genetic evidence for causal association between atrial fibrillation and dementia: A Mendelian randomization study. J Am Heart Assoc. 2023;12:e029623. 37548160 10.1161/JAHA.123.029623 PMC10492936

[38] Pan Y , Wang Y, Wang Y. Investigation of causal effect of atrial fibrillation on Alzheimer disease: A Mendelian randomization study. J Am Heart Assoc. 2020;9(2):e014889. 31914880 10.1161/JAHA.119.014889 PMC7033843

[39] Li Q , Yan S, Li Y, Kang H, Zhu H, Lv C. Mendelian randomization study of heart failure and stroke subtypes. Front Cardiovasc Med. 2022;9:844733. 35463787 10.3389/fcvm.2022.844733 PMC9021833

[40] Zhang L , Liu W, Sun W, et al Heart failure and ischemic stroke: A bidirectional and multivariable Mendelian randomization study. Front Genet. 2021;12:771044. 34912375 10.3389/fgene.2021.771044 PMC8666512

[41] Arega Y , Shao Y. Heart failure and late-onset Alzheimer’s disease: A Mendelian randomization study. Front Genet. 2022;13:1015674. 36523758 10.3389/fgene.2022.1015674 PMC9745072

[42] Lubitz SA , Parsons OE, Anderson CD, et al Atrial fibrillation genetic risk and ischemic stroke mechanisms. Stroke. 2017;48(6):1451–1456. 28468926 10.1161/STROKEAHA.116.016198 PMC7438180

[43] Malik R , Bevan S, Nalls MA, et al Multilocus genetic risk score associates with ischemic stroke in case-control and prospective cohort studies. Stroke. 2014;45(2):394–402. 24436234 10.1161/STROKEAHA.113.002938 PMC4006951

[44] Tada H , Shiffman D, Smith JG, et al Twelve-single nucleotide polymorphism genetic risk score identifies individuals at increased risk for future atrial fibrillation and stroke. Stroke. 2014;45(10):2856–2862. 25123217 10.1161/STROKEAHA.114.006072 PMC4346099

[45] Rydén L , Wetterberg H, Ahlner F, et al Attrition in the Gothenburg H70 birth cohort studies, an 18-year follow-up of the 1930 cohort. Front Epidemiol. 2023;3:1151519. 38455909 10.3389/fepid.2023.1151519 PMC10910926

[46] Wetterberg H , Rydén L, Ahlner F, et al Representativeness in population-based studies of older adults: Five waves of cross-sectional examinations in the Gothenburg H70 birth cohort study. BMJ Open. 2022;12(12):e068165. 10.1136/bmjopen-2022-068165 PMC9764666 36526314

[47] Folstein MF , Folstein SE, McHugh PR. “Mini-mental state”. A practical method for grading the cognitive state of patients for the clinician. J Psychiatr Res. 1975;12(3):189–198. 1202204 10.1016/0022-3956(75)90026-6

[48] Socialstyrelsen . Historik om patientregistret. 2023-11-30, 2023. Updated 2020-03-06. Accessed 2023-11-30, 2023. https://www.socialstyrelsen.se/statistik-och-data/register/patientregistret/historik/

[49] Ludvigsson JF , Andersson E, Ekbom A, et al External review and validation of the Swedish national inpatient register. BMC Public Health. 2011;11:450. 21658213 10.1186/1471-2458-11-450 PMC3142234

[50] Blauwendraat C , Faghri F, Pihlstrom L, et al NeuroChip, an updated version of the NeuroX genotyping platform to rapidly screen for variants associated with neurological diseases. Neurobiol Aging. 2017;57:247.e9–247.e13. 10.1016/j.neurobiolaging.2017.05.009 PMC5534378 28602509

[51] Nielsen JB , Thorolfsdottir RB, Fritsche LG, et al Biobank-driven genomic discovery yields new insight into atrial fibrillation biology. Nat Genet. 2018;50(9):1234–1239. 30061737 10.1038/s41588-018-0171-3 PMC6530775

[52] Christophersen IE , Rienstra M, Roselli C, et al Large-scale analyses of common and rare variants identify 12 new loci associated with atrial fibrillation. Nat Genet. 2017;49(6):946–952. 28416818 10.1038/ng.3843 PMC5585859

[53] Shah S , Henry A, Roselli C, et al Genome-wide association and Mendelian randomisation analysis provide insights into the pathogenesis of heart failure. Nat Commun. 2020;11(1):163. 31919418 10.1038/s41467-019-13690-5 PMC6952380

[54] Wetterberg H , Najar J, Rydberg Sterner T, et al Decreasing incidence and prevalence of dementia among octogenarians: A population-based study on 3 cohorts born 30 years apart. J Gerontol A Biol Sci Med Sci. 2023;78(6):1069–1077. 36843145 10.1093/gerona/glad071 PMC10235204

[55] de Bruijn RF , Heeringa J, Wolters FJ, et al Association between atrial fibrillation and dementia in the general population. JAMA Neurol. 2015;72(11):1288–1294. 26389654 10.1001/jamaneurol.2015.2161

[56] Ding M , Fratiglioni L, Johnell K, et al Atrial fibrillation, antithrombotic treatment, and cognitive aging: A population-based study. Neurology. 2018;91(19):e1732–e1740. 30305443 10.1212/WNL.0000000000006456 PMC6251601

[57] Dublin S , Anderson ML, Haneuse SJ, et al Atrial fibrillation and risk of dementia: A prospective cohort study. J Am Geriatr Soc. 2011;59(8):1369–1375. 21806558 10.1111/j.1532-5415.2011.03508.x PMC3289545

[58] Chen LY , Norby FL, Gottesman RF, et al Association of atrial fibrillation with cognitive decline and dementia over 20 years: The ARIC-NCS (atherosclerosis risk in communities neurocognitive study). J Am Heart Assoc. 2018;7(6):e007301. 29514809 10.1161/JAHA.117.007301 PMC5907543

[59] Singh-Manoux A , Fayosse A, Sabia S, et al Atrial fibrillation as a risk factor for cognitive decline and dementia. Eur Heart J. 2017;38(34):2612–2618. 28460139 10.1093/eurheartj/ehx208 PMC5837240

[60] Kim D , Yang PS, Lip GYH, Joung B. Atrial fibrillation increases the risk of early-onset dementia in the general population: Data from a population-based cohort. J Clin Med. 2020;9(11):3665. 33202611 10.3390/jcm9113665 PMC7697737

[61] Hu YF , Chen YJ, Lin YJ, Chen SA. Inflammation and the pathogenesis of atrial fibrillation. Nat Rev Cardiol. 2015;12(4):230–243. 25622848 10.1038/nrcardio.2015.2

[62] Troncone L , Luciani M, Coggins M, et al Aβ amyloid pathology affects the hearts of patients with Alzheimer’s disease: Mind the heart. J Am Coll Cardiol. 2016;68(22):2395–2407. 27908343 10.1016/j.jacc.2016.08.073 PMC5142757

[63] Pulit SL , Weng LC, McArdle PF, et al Atrial fibrillation genetic risk differentiates cardioembolic stroke from other stroke subtypes. Neurol Genet. 2018;4(6):e293. 30584597 10.1212/NXG.0000000000000293 PMC6283455

[64] Kamel H , Okin PM, Elkind MS, Iadecola C. Atrial fibrillation and mechanisms of stroke: Time for a new model. Stroke. 2016;47(3):895–900. 26786114 10.1161/STROKEAHA.115.012004 PMC4766055

[65] Vishwanath S , Qaderi V, Steves CJ, Reid CM, Hopper I, Ryan J. Cognitive decline and risk of dementia in individuals with heart failure: A systematic review and meta-analysis. J Card Fail. 2022;28(8):1337–1348. 34971812 10.1016/j.cardfail.2021.12.014

[66] Jefferson AL , Beiser AS, Himali JJ, et al Low cardiac index is associated with incident dementia and Alzheimer disease: The Framingham heart study. Circulation. 2015;131(15):1333–1339. 25700178 10.1161/CIRCULATIONAHA.114.012438 PMC4398627

[67] Cermakova P , Eriksdotter M, Lund LH, Winblad B, Religa P, Religa D. Heart failure and Alzheimer’s disease. J Intern Med. 2015;277(4):406–425. 25041352 10.1111/joim.12287 PMC4409079

[68] White M , Ducharme A, Ibrahim R, et al Increased systemic inflammation and oxidative stress in patients with worsening congestive heart failure: Improvement after short-term inotropic support. Clin Sci (Lond). 2006;110(4):483–489. 16402915 10.1042/CS20050317

[69] Duan C , Shi J, Yuan G, et al Causal association between heart failure and Alzheimer’s disease: A two-sample bidirectional Mendelian randomization study. Front Genet. 2021;12:772343. 35087565 10.3389/fgene.2021.772343 PMC8787319

[70] Lip GY , Gibbs CR. Does heart failure confer a hypercoagulable state? Virchow’s triad revisited. J Am Coll Cardiol. 1999;33(5):1424–1426. 10193748 10.1016/s0735-1097(99)00033-9

[71] Rizzuto D , Feldman AL, Karlsson IK, Dahl Aslan AK, Gatz M, Pedersen NL. Detection of dementia cases in two Swedish health registers: A validation study. J Alzheimers Dis. 2018;61(4):1301–1310. 29376854 10.3233/JAD-170572 PMC6218116

